# Curcumin Potentiates α7 Nicotinic Acetylcholine Receptors and Alleviates Autistic-Like Social Deficits and Brain Oxidative Stress Status in Mice

**DOI:** 10.3390/ijms22147251

**Published:** 2021-07-06

**Authors:** Petrilla Jayaprakash, Dmytro Isaev, Waheed Shabbir, Dietrich E. Lorke, Bassem Sadek, Murat Oz

**Affiliations:** 1 Department of Pharmacology & Therapeutics, College of Medicine and Health Sciences, UAE University, Abu Dhabi P.O. Box 17666, United Arab Emirates; petrilla.jp@uaeu.ac.ae (P.J.); dmytro.isaev@gmail.com (D.I.); 2 Zayed Center for Health Sciences, United Arab Emirates University, Abu Dhabi P.O. Box 17666, United Arab Emirates; 3 Department of Cellular Membranology, Bogomoletz Institute of Physiology, 1024 Kiev, Ukraine; 4 Department of Pharmacology and Toxicology, University of Vienna, 1010 Vienna, Austria; waheed.shabbir@ucsf.edu; 5 Department of Medicine, Division of Nephrology and Cellular and Molecular Pharmacology, University of California, San Francisco, CA 94158-2140, USA; 6 Department of Anatomy and Cellular Biology, College of Medicine and Health Sciences, Khalifa University, Abu Dhabi P.O. Box 127788, United Arab Emirates; dietrich.lorke@ku.ac.ae; 7 Department of Pharmacology and Therapeutics, Faculty of Pharmacy, Kuwait University, Safat 13110, Kuwait

**Keywords:** autism spectrum disorder, nicotinic receptors, curcumin, positive allosteric modulator, social features, oxidative stress, BTBR mice

## Abstract

Autistic spectrum disorder (ASD) refers to a group of neurodevelopmental disorders characterized by impaired social interaction and cognitive deficit, restricted repetitive behaviors, altered immune responses, and imbalanced oxidative stress status. In recent years, there has been a growing interest in studying the role of nicotinic acetylcholine receptors (nAChRs), specifically α7-nAChRs, in the CNS. Influence of agonists for α7-nAChRs on the cognitive behavior, learning, and memory formation has been demonstrated in neuro-pathological condition such as ASD and attention-deficit hyperactivity disorder (ADHD). Curcumin (CUR), the active compound of the spice turmeric, has been shown to act as a positive allosteric modulator of α7-nAChRs. Here we hypothesize that CUR, acting through α7-nAChRs, influences the neuropathology of ASD. In patch clamp studies, fast inward currents activated by choline, a selective agonist of α7-nAChRs, were significantly potentiated by CUR. Moreover, choline induced enhancement of spontaneous inhibitory postsynaptic currents was markedly increased in the presence of CUR. Furthermore, CUR (25, 50, and 100 mg/kg, i.p.) ameliorated dose-dependent social deficits without affecting locomotor activity or anxiety-like behaviors of tested male Black and Tan BRachyury (BTBR) mice. In addition, CUR (50 and 100 mg/kg, i.p.) mitigated oxidative stress status by restoring the decreased levels of superoxide dismutase (SOD) and catalase (CAT) in the hippocampus and the cerebellum of treated mice. Collectively, the observed results indicate that CUR potentiates α7-nAChRs in native central nervous system neurons, mitigates disturbed oxidative stress, and alleviates ASD-like features in BTBR mice used as an idiopathic rodent model of ASD, and may represent a promising novel pharmacological strategy for ASD treatment.

## 1. Introduction

Nicotinic acetylcholine (nACh) receptors belong to the ligand-gated ion channel family that includes GABAA, glycine, and 5-HT3 receptors. The homomeric α7-nACh receptor subtype is abundantly present in the central nervous system (CNS) and periphery and plays a key role in synaptic plasticity and various disease pathologies [1,2]. In recent years, there has been a growing interest in studying the role of α7-nACh receptors (α7-nAChRs) in the CNS. The influence of α7-nAChRs on cognitive behavior, learning, and memory formation has been demonstrated in both physiological and neuro-pathological conditions, including Alzheimer’s disease [2,3,4], cognitive deficits [5], and schizophrenia [6]. Importantly, a growing body of literature implicates the α7-nAChRs in the pathobiology of autistic spectrum disorder (ASD) [7,8] which refers to a group of neurodevelopmental disorders characterized by impaired social interaction and memory, restricted repetitive behaviors, and attention deficit hyperactivity disorder (ADHD) [9,10]. The role of α7-nAChRs in the pathogenesis of ASD has been investigated in several in vitro, in vivo, and clinical studies. Early observations showed that there were larger and increased number of neurons in the basal forebrain (a site of origin of cholinergic projections in the CNS) of children with ASD, whereas there were smaller and fewer neurons in adults with ASD, relative to controls [9], suggesting functional disruption of cholinergic transmission in patients diagnosed with ASD. In addition, binding of α-bungarotoxin, a marker for α7-containing nAChR subtypes, is increased in the cerebellum [11,12], while α7 immunobinding is decreased in the thalamus of autistic individuals [10]. These alterations of the cholinergic system in patients diagnosed with ASD appear to specifically involve nAChRs since markers of cholinergic synaptic transmission (choline acetyltransferase and acetylcholinesterase activity) remained unaltered [11,12] and muscarinic receptors remained unchanged [10,11,12]. Furthermore, there is evidence that cotinine, the primary metabolite of nicotine and a positive allosteric modulator of α7-nAChR, improves impaired cognition in the mouse model of Fragile X syndrome, a condition that is strongly associated with ASD [13]. Electrophysiological studies have demonstrated significant (64%) decrease of α7-nAChR-mediated whole-cell currents and decreased expression of α7-nAChR in locus coeruleus neurons of Mccp2-null mice, an animal model for ASD that results from mutations to the X-linked gene [14]. Also, pharmacological administration of choline, a selective α7-nAChR agonist, beginning in early pregnancy and continuing throughout gestation and lactation has been shown to attenuate some of the deleterious ASD-like effects of maternal immune activation, an in vivo model for ASD, on the development of the offspring [15]. Moreover, an exploratory clinical trial of transdermal nicotine for aggression and irritability in adults with ASD showed that irritability and sleep ratings were improved by nicotine compared to the placebo group, supporting further investigation of nAChR agonists for aggression and sleep in ASD [16]. Furthermore, mutation of the human chromosome 15q13.3 was found to increase the risk for ASD and schizophrenia. One of the noteworthy genes in 15q13.3 is CHRNA7, which encodes the α7-nAChR, associated with ASD and cognitive deficits in clinical studies and rodent models [17,18,19,20,21]. In addition, significant reductions of the expression of CHRNA7 have been shown in the frontal cortex of individuals diagnosed with Rett syndrome, a neurodevelopmental disorder strongly associated with ASD [22]. Also, the brain of the BTBR mouse, a well-studied idiopathic ASD model shown to display core ASD phenotypes (decreased sociability, altered vocalization, and repetitive behavior) [23,24], has decreased ACh levels and increased levels of kynurenic acid, a α7-nAChR antagonist, in medial prefrontal cortex [25]. Notably, decreased social interaction and repetitive behaviors, such as self-grooming, was significantly attenuated by the administration of α7-nAChR agonist, AVL-3288 [26], and nicotine [5,27] in BTBR mice. In clinical trials, the acetylcholinesterase inhibitor galantamine, an acetylcholinesterase inhibitor (AChEI), and positive allosteric modulator of α7-nAChR, showed beneficial effects in ASD individuals [28,29]. Another AChEI, namely donepezil (DOZ), increased rapid eye movement sleep time in ASD children, which is thought to be a salutary effect in ASD patients [30]. 

**Figure 1:**
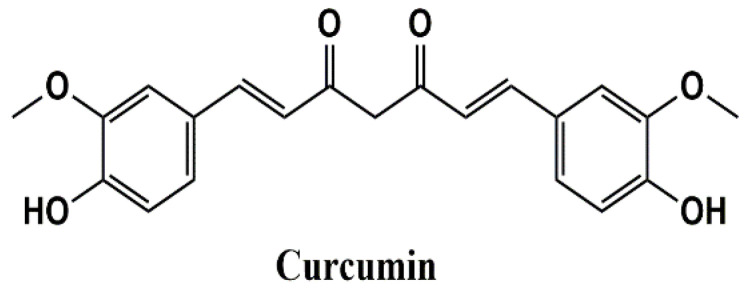
Chemical structure of curcumin, the active compound of the spice turmeric.

Curcumin (CUR), the active compound of the spice turmeric (Figure 1), has been used for ages in Ayurvedic and Chinese medicine to treat a wide range of ailments. Extensive research over the past half century has shown that CUR has anti-inflammatory, anti-oxidant, and neuroprotective effects [31,32,33,34]. In earlier studies CUR was identified as a positive allosteric modulator of α7-nAChRs expressed in *Xenopus oocytes* [35,36], mammalian cell lines [35,37], and in vivo studies [38]. 

Therefore, the present study aimed to investigate the in vitro effects of CUR on α7-nAChRs in hippocampal neurons. Moreover, the in vivo effects of chronic systemic administration of CUR on ASD- and anxiety-like behaviors in male Black and Tan BRachyury (BTBR) as an idiopathic ASD rodent model were assessed. Furthermore, the mitigating effects of CUR on oxidative stress levels in the hippocampus and the cerebellum of treated animals were tested. To comprehend our results, the ability of methyllycaconitine (MLA) to reverse the CUR provided effects were evaluated to elucidate the possible involvement of α7-nAChRs and ACh in the effects observed for CUR. The in vitro effects observed for CUR in the current study were in neurons of rats, but there were only BTBR mice available as idiopathic animal model of ASD-like features to carry out our behavioral as well as biochemical assessments. The discrepancy in animal species used in the current study will not affect our whole hypothesis as previous studies revealed that mouse neuronal α7-AChR, which is known to bind to α-bungarotoxin, a selective α7-nAChR marker, in mammalian brain, shows substantial identity to the rat (99.6%) and human (92.8%) amino acid sequences [39].

## 2. Results

### 2.1. Effects of CUR on α7-nACh Receptors in the CA1 Region of Stratum Radiatum Interneurons in Rat Hippocampal Brain Slices 

**Figure 2:**
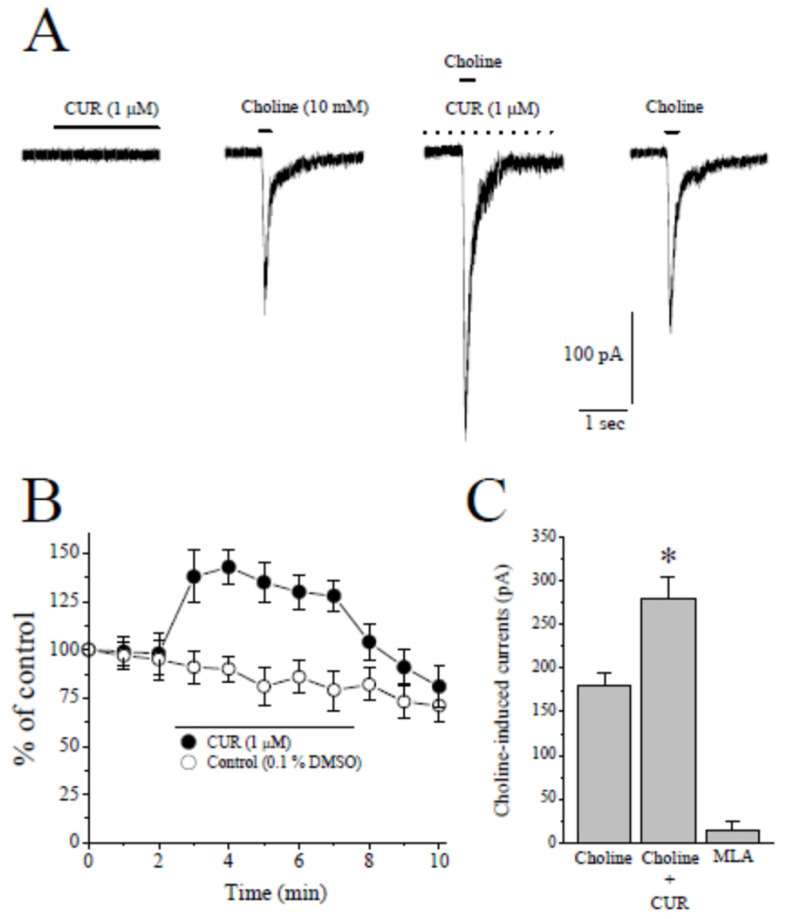
The effect of curcumin on choline-induced ion currents recorded in CA1 area stratum radiatum interneurons of rat hippocampal slices. (**A**) Recordings of holding current in the presence of curcumin for 30 s (on the left), choline-induced currents before (control, second panel from left), during (5 min of curcumin) and after (2 min of recovery) the bath application of 1 µM curcumin in hippocampal interneurons. Choline application was represented with a short solid bar on top of the current traces. The dashed line indicates continuing bath application of curcumin. (**B**) Time-course of the effect of vehicle (0.1% DMSO; open circles) and curcumin (1 µM; filled circles) on the peaks of the Choline-induced currents. Each data point represents the normalized mean ± S.E.M. of five to seven experiments. The duration of drug application is indicated by the horizontal bar in the figure. (**C**) Summary of the effects of curcumin and methyllycaconitine on the peak amplitudes of choline induced currents. Bars represent the means ± S.E. of four to eight experiments (* *p* < 0.05 vs. control; ANOVA). MLA, methyllycaconitine.

In whole cell patch clamp mode, focal application of 10 mM choline, a selective agonist for α7-nACh receptor [1], caused rapidly activating and fast desensitizing inward currents that were completely inhibited by the bath application of 10 µM MLA, a selective antagonist for α7-nACh receptor (*n* = 4). Choline-induced currents were significantly potentiated by 5 min bath application of 1 µM CUR (Figure 2A and Figure S1). Time-courses of effects of CUR and the vehicle applications on the amplitudes of choline-induced currents are presented in Figure 2B. CUR (1 μM) caused a significant potentiation of the current which was partially reversed during 5 min washout period. In the absence of curcumin, vehicle (0.1% DMSO) alone did not alter the amplitude of the choline-induced current (Figure 2B, controls). Summary of the effect of CUR and MLA was presented in Figure 2C.

### 2.2. Effects of CUR on Choline-Induced GABA Responses in CA1 Pyramidal Neurons of Hippocampal Slices 

**Figure 3:**
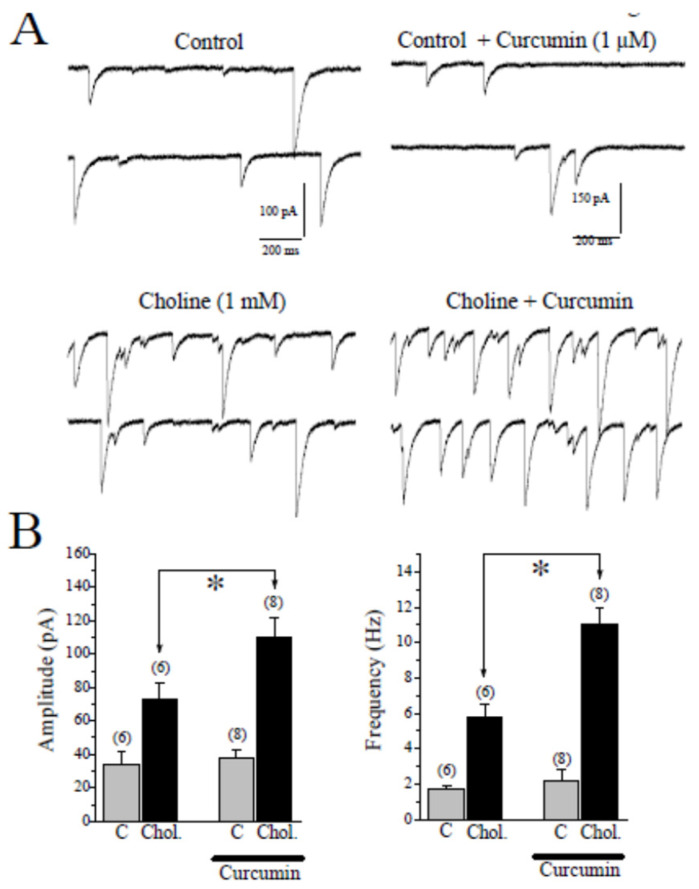
The effect of curcumin on choline-induced enhancement of GABAA receptor-mediated spontaneous synaptic events in CA1 pyramidal neurons. Whole-cell recordings were performed using CsCl-based electrode solution at a holding potential of −70 mV. (**A**) On the left, the application of choline (2 mM) for 30 s increased the amplitudes and frequencies of spontaneous inhibitory postsynaptic currents (sIPSCs; *n* = 6). On the right, in another cell, choline-induced enhancements of sIPSCs were increased significantly after 2 min preincubation in curcumin (1 µM). (**B**) Summary of the effects of curcumin (1 µM) on choline-induced responses. The averaged amplitudes (on the left) and the frequencies (on the right) of sIPSCs were presented before (control **C**, gray bars) and after (black bars) choline (Chol. 2 mM) application. For comparison, the effect of choline on the GABAA receptor-mediated sIPSCs is shown in the absence and the presence of curcumin. Bars represent the means ± S.E. of six to eight experiments (* *p* < 0.05 vs. control; ANOVA). C, control; Chol., choline.

In the hippocampus, α7-nAChRs are located on both GABAergic and glutamatergic interneurons and application of choline, a selective agonist for α7-nAChRs, increases both excitatory and inhibitory inputs to CA1 pyramidal neurons [1]. For this reason, we have isolated spontaneously occurring GABAA receptor-mediated synaptic currents (sIPSCs) by pharmacological means (DL-2-amino-5-phosphonovalerate (APV) plus 6,7-dinitroquinoxaline-2,3-dione (DNQX)) and recorded choline-induced GABA responses in CA1 pyramidal neurons of hippocampal slices. Thereafter the effects of choline were assessed in the absence and presence of 1 µM CUR. Application of choline (2 mM for 30 s) caused a transient increase in amplitudes and frequencies of sIPSCs that lasted for 1–2 min (Figure 3A,B; *n* = 6). Choline-induced increases in sIPSCs were completely abolished by 1 min pre-application of 10 µM MLA (*n* = 4, data shown in Supplement Figure S1), indicating that the effect is mediated by activation of the α7-nAChR. Two min pretreatment with 1 μM CUR markedly increased the effects of choline on the amplitudes and the frequencies of sIPSCs in 8/8 pyramidal neurons (Figure 3B). When miniature IPSCs (mIPSCs) were measured in the presence of 1 µM TTX, CUR (1 μM, 5 min) alone did not change the amplitudes and the frequencies of mIPSCs (*n* = 5). The amplitudes and frequencies of mIPSCs in the presence and absence of CUR were 17.1 ± 2.3 pA and 0.7 ± 0.3 Hz (*n* = 4), and 19.6 ± 1.8 pA and 0.8 ± 0.2 Hz (*n* = 5), respectively. There were no statistically significant differences between control and CUR treated groups with respect to means of amplitudes and frequencies (*p* > 0.05, ANOVA, *n* = 5), suggesting that postsynaptic GABAA receptors are not affected by application of CUR.

### 2.3. Effects of CUR and DOZ on Sociability and Social Novelty Deficits of BTBR Mice in TCP

**Figure 4:**
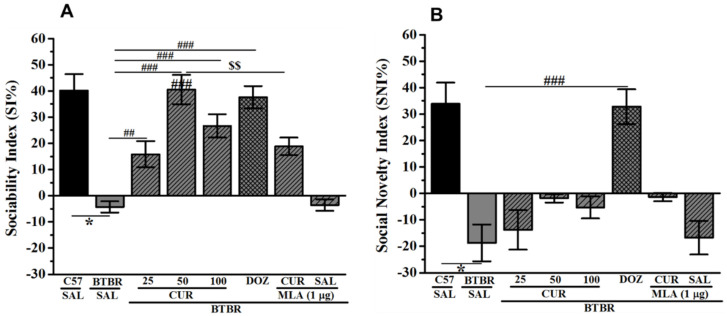
Effects of curcumin and donepezil on deficits of sociability and social novelty preference in BTBR mice. After 10 min of habituation, male subjects were allowed to explore all chambers for two 10 min sessions. C57 and BTBR mice were injected with saline, and BTBR mice were administered with CUR (25, 50, or 100 mg/kg, i.p.) or DOZ (1 mg/kg, i.p.) chronically for 21 days. The results obtained were Sociability Index (SI) and Social Novelty Index (SNI) (B). Also, the effects of chronic (21 days) systemic co-injection of MLA (1 µg/kg, i.p.) on the CUR-(50 mg)-provided improvement of sociability (**A**) and social novelty preference (**B**) were assessed. Figures show mean ± SEM (*n* = 7). * *p* < 0.05 vs. SI or SNI of saline-treated C57 mice. ^##^ *p* < 0.01 vs. SI of saline-treated BTBR mice. ^###^ *p* < 0.001 vs. SI or SNI of saline-treated BTBR mice. ^$$^ *p* < 0.05 vs. CUR-(50 mg)-treated BTBR mice.

The effects of chronic systemic administration of CUR (25, 50, and 100 mg/kg, i.p.) and DOZ (1 mg/kg, i.p.) on ASD-like sociability and social novelty deficits in the TCP task in BTBR mice are displayed in Figure 4A,B. Statistical analyses revealed that chronic pretreatment with CUR (50 mg/kg, i.p.) or DOZ (1 mg/kg) prior to TCP increased SI significantly (Figure 4A). Post hoc analysis showed that BTBR mice displayed significant sociability deficits expressed in form of SI value than SI values of control animals, with (*F*_(1,12)_ = 13.30; *p* < 0.05) (Figure 4A). However, CUR (25, 50, and 100 mg/kg) or DOZ (1 mg/kg) significantly increased SI of BTBR mice when compared to saline treated BTBR mice group, with (*F*_(1,12)_ = 11.78; *p* < 0.01), (*F*_(1,12)_ = 47.97; *p* < 0.001), (*F*_(1,12)_ = 33.63; *p* < 0.001), and (*F*_(1,12)_ = 65.63; *p* < 0.001), respectively (Figure 4A). Moreover, the results revealed that the enhancement in SI observed with CUR (50 mg/kg) was comparable to that shown with DOZ (1 mg/kg), with (*F*_(1,12)_ = 0.15; *p* = 0.70) (Figure 4A). Interestingly, chronic systemic co-administration with the brain penetrant selective α7-nACh receptor antagonist MLA (1 µ mg/kg, i.p.) reversed the CUR (50 mg)-provided sociability improvement, with (*F*_(1,12)_ = 9.47; *p* < 0.05) (Figure 4A). Notably, chronic systemic pretreatment of BTBR mice with MLA (1 µ mg/kg, i.p.) did not change the SI value of tested control animals, with (*F*_(1,12)_ = 0.05; *p* = 0.83) (Figure 4A). The statistical analyses of the results exhibited that chronic pretreatment with CUR (25, 50 and 100 mg/kg, i.p.) prior to TCP failed to enhance social novelty preference measured by assessing the SNI values (*p*’s > 0.05) (Figure 4B). However, improvement in social novelty preference was achieved following chronic systemic pretreatment of BTBR mice with the reference drug DOZ (1 mg/kg, i.p.), with (*F*_(1,12)_ = 24.70; *p* < 0.001) (Figure 4B). In addition, chronic systemic pretreatment of control BTBR mice with MLA (1 µ mg/kg, i.p.) did not change the SNI value of tested control animals, with (*F*_(1,12)_ = 0.04; *p* = 0.85) (Figure 4A). 

### 2.4. Effects of CUR and DOZ on Locomotor Activity and Anxiety Levels in OFT

The findings observed for locomotor activity in C57 and BTBR mice are shown in Table 1. For the total distance travelled, there was a significant effect of strain (*p* < 0.01), but there was no significant effect for treatment or strain x treatment interaction (*p*’s > 0.05) (Table 1). Post hoc tests revealed that vehicle-treated BTBR showed a significant increase in the distance travelled when compared with vehicle-treated C57 mice, with values of (*F*_(1,12)_ = 38.95, *p* < 0.001). C57 and BTBR mice pretreated with CUR (25, 50, and 100 mg/kg, i.p.), DOZ (1 mg/kg, i.p.), or MLA (1 µg/kg, i.p.) did not show any alteration in the total distance travelled (all *p*’s > 0.05) (Table 1). The observed results revealed that chronic systemic injection of CUR (25, 50, or 100 mg/kg, i.p.) failed to alter time spent in the center of the arena or in the periphery (all *p*’s > 0.05) (Table 1). However, DOZ (1 mg/kg, i.p.) significantly mitigated the increased time spent in the center of the arena, with (*F*_(1,12)_ = 11.58; *p* < 0.05) (Table 1). Interestingly and for the time spent in the center and in the periphery, there was no significant effect of strain (*p* > 0.05), but there was significant effect for treatment and the strain x treatment interaction, with values of (all *p*’s < 0.01).

### 2.5. Effects of CUR and DOZ on Levels of Oxidative Stress Markers in the Hippocampus and the Cerebellum of Treated BTBR Mice

Superoxide dismutase (SOD) and catalase (CAT) were assessed in two different brain parts of treated BTBR mice, namely the cerebellum and the hippocampus. BTBR mice showed a significant decrease in SOD and CAT compared to C57 mice (Table 2). Effects of systemic administration of CUR (25, 50, and 100 mg/kg, i.p.) was assessed in C57 and BTBR mice. Statistical analysis showed that SOD and CAT were significantly reduced in both brain parts of BTBR mice compared to C57 mice (all *P*’s < 0.05) (Table 2). CUR (50 and 100 mg/kg, i.p.) significantly increased the reduced levels of SOD in the hippocampus (all *p*’s < 0.05), but failed to increase the decreased levels of SOD in the cerebellum of treated BTBR mice. Moreover, CUR (50 and 100 mg/kg, i.p.) significantly increased the reduced levels of CAT in the cerebellum (all *p*’s < 0.05), but failed to increase the decreased levels of CAT in the hippocampus. Notably, chronic systemic administration with reference drug DOZ (1 mg/kg, i.p.) failed to mitigate both enzymes in hippocampal and cerebellar brain tissues of treated BTBR mice (Table 2). 

## 3. Discussion

The results indicate that CUR potentiates α7-nAChRs in native neurons of CNS and alleviates autism-like features in rodent models by enhancing sociability of tested BTBR mice as an idiopathic rodent model for ASD. The role of cholinergic neurotransmission is crucial in ASD-like features of BTBR mice, as recent preclinical studies revealed that deficit in cholinergic neurotransmission and low levels of brain ACh are present in this species of rodents [25]. In addition to the well-established positive effect of AChEIs on cognition in the wealth of studies, treatment with the standard drug DOZ was reported by Reidel et al. to relieve social memory deficiency [40]. This evidence fits with our findings observed for CUR and DOZ in the current series of behavioral experiments. Accordingly, CUR and DOZ improved the social deficits of pretreated BTBR mice. Moreover, the results observed for CUR are in agreement with a previous preclinical study in which chronic systemic treatment with CUR for a duration of four weeks was found to significantly and dose-dependently restore neurological, behavioral, biochemical, and molecular changes associated with autistic phenotype in experimental rodents [41]. The observed effects for CUR in sociability assessment were completely nullified when MLA was co-administered, indicating that cholinergic neurotransmission through α7-nAChRs may be involved in the effects observed for CUR on the sociability parameters of tested animals. Notably, our results observed for CUR (25, 50, and 100 mg/kg) on social deficits adapted an inverted U shaped effect of CUR when used at different doses. The latter findings are in harmony with previously described U shaped dose–response relationships of nicotine on α7-nAChRs in behavioral studies [42,43,44,45]. Furthermore, a significant choline deficiency, a precursor molecule for the synthesis of ACh neurotransmitter and α7-nAChR agonist, was reported in the brains of persons with ASD [46]. Also, alterations in the levels of α7-nAChRs were observed in numerous areas of the brain, including neocortex, thalamus, striatum, and cerebellum, of ASD individuals, with the central abnormality being the diminishment of muscarinic receptors (M1 subtype) [46,47,48]. Interestingly, CUR has been shown to have a beneficial effect in numerous previous rodent models of ASD [41,49]. Notably, the concentration of CUR in plasma and its ability to pass the blood–brain barrier following oral and intravenous administration has been studied previously [49]. When CUR is given orally at a dose of 2 g/kg to rats, maximum serum concentration of 1.35 µg/mL (3.5 µM) was attained. Since CUR is a highly lipophilic compound with LogP (octanol–water partition coefficient) value of 3.3 [50], its membrane concentration is expected to be considerably higher than blood levels. Therefore, the functional modulation of α7-nAChRs demonstrated in this study can be pharmacologically relevant. Our results confirm these studies and indicate that potentiation of α7-nAChR plays a role in beneficial effects of CUR in autistic rodents, as co-administration with the selective α7-nAChR antagonist MLA reversed the CUR (50 mg)-provided sociability enhancement of tested BTBR mice. Interestingly, a previous study revealed that DOZ showed an antagonistic effect at α7-nAChR in rat neurons [51]. However, in that study the antagonistic effect of DOZ was achieved with a brain concentration of 100 µM, a concentration that requires 1.5 mg/kg or higher to be administered to achieve such high brain level of DOZ [52]. In the current series of behavioral experiments, DOZ was used at a dose of 1 mg/kg, excluding the possibility that such high brain level of 100µM would have been achieved, a level capable of inducing this α7-nAChR antagonism. Furthermore, the reference DOZ (1 mg/kg, i.p.) was found to totally restore the abnormal anxiety levels of treated BTBR mice in OFT. However, CUR (25, 50, 100 mg/kg) failed to alter the abnormal anxiety-like features of tested mice. Importantly, CUR (25, 50, 100 mg/kg, i.p.) and DOZ failed to alter time spent in the periphery and total distance travelled of treated BTBR mice, excluding any confounding factors, such as alteration of locomotor activity, which may provide false positive observations in regards to the CUR-provided improvements on social features. Another main objective of the current study was to assess the capability CUR (25, 50, and 100 mg/kg) to mitigate disturbed levels of oxidative stress in two different brain areas of treated BTBR mice, namely the cerebellum and the hippocampus. In the current study, the results showed that BTBR mice with ASD-like features displayed significant decreases in SOD and CAT levels in both assessed brain parts of tested BTBR mice and as compared to control mice. Systemic chronic administration of CUR (50 and 100 mg/kg) restored the levels of CAT in hippocampal tissues, but failed to do so in cerebellar tissues of treated BTBR mice. Contrary, CUR (25, 50, and 100 mg/kg) were capable of restoring the levels of CAT levels in the cerebellum, but failed to alter the levels of SOD in the hippocampus. The results observed on the levels of oxidative stress markers are in harmony with numerous previous preclinical reports from our group and other research groups that investigated the effects of synthetic ligands modulating brain ACh levels on oxidative stress in the brains of different rodent models [53,54,55,56,57]. Also, the results observed for CUR are in harmony with a previous preclinical study in which CUR was found to mitigate the disturbed levels of IFN-γ, serotonin, glutamine, reduced glutathione, glutathione *S*-transferase, lipid peroxidase with an increase in CYP450, IL-6, glutamate, and oxidized glutathione in valproate-induced ASD in rats [58]. However, one of the significant limitations of the current findings is that more detailed studies are still needed to assess the pharmacokinetic parameters of CUR in BTBR mice and also to mechanistically explain the effects of CUR on electrophysiology recordings, social features in tested mice, and oxidative stress enzymes SOD and CAT, considering that CUR targets α7-nAChRs. Moreover, further pharmacokinetic assessments are warranted to evaluate the central bioavailability of CUR and its distribution/metabolism.

## 4. Materials and Methods

### 4.1. Recordings from Hippocampal Slices 

Horizontal slices (300–350 μm) prepared from 15- to 30-day old male Sprague Dawley rats using a vibratome (Pelco, Redding, CA, USA), as described earlier [59,60]. Slices were incubated at 30 °C for 30 min and then maintained submerged at room temperature. The artificial cerebral spinal fluid (ACSF) for cutting and incubating slices contained (in mM): 124 NaCI, 2.5 KCI, 1.2 NaH2PO_4_, 2.5 MgSO_4_, 10 D-glucose, 1 CaCI_2_, and 25.9 NaHCO_3_, saturated with 95% O_2_/5% CO_2_. Following at least one-hour incubation, slices were transferred to a recording chamber where they were superfused at a rate of 5 mL/min with ACSF that was heated to 30 °C. Whole-cell patches from the somata of CA1 area interneurons were made under visual control using upright microscope (Nikon Eclipse E600 FN, Garden City, NY, USA) equipped with infrared differential interference contrast video microscopy system and 40× water immersion objective lens. Whole-cell patch-clamp recordings were made with pipettes pulled on a Flaming/Brown electrode puller (Sutter Instruments, Novato, CA, USA). Pipettes are typically 3–5 MΩ when filled with an internal solution that contains (in mM): 140 Cs-MeSO_3_, 4 NaCI, 1 MgCI_2_, 0.2 EGTA, 10 HEPES, 2 MgATP, and 0.3 Na3GTP (pH 7.3 using additional CsOH or KOH and volume adjusted to 285 mOsm). Cells were voltage clamped using an Axopatch 200B amplifier (Axon Instruments, Sunnyvale, CA, USA) at a holding potential of −70 mV. A picospritzer (General Valve, Fairfield, NJ, USA) were used to apply choline (10 mM; for 100 ms) from pipettes identical to those used for whole-cell recording. Atropine (1 µM), a muscarinic receptor antagonist and TTX (1 µM), an inhibitor of voltage-gated Na+ channels, were routinely included in extracellular solution. Data were sampled at 20 kHz, filtered at 2 kHz, recorded on a computer via a Digidata 1321 analogue-digital converter using Clampex (Axon Instruments, Sunnyvale, CA, USA), and analyzed using Clampfit (Axon Instruments, CA, USA), and OriginPro v8.5 (OriginLab, Boston, MA, USA). All chemicals were purchased from Sigma (St. Louis, MO, USA). 

Spontaneous inhibitory postsynaptic currents were recorded in CA1 pyramidal neurons as described before [61]. Cells were voltage clamped at 70 mV using whole-cell electrodes containing (in mM): CsCl 125.0, HEPES 10.0, EGTA 1.0, CaCl2 0.1, Mg2 + −ATP 2.0, Na + −GTP 0.2, and the quaternary lidocaine derivative QX-314 2, pH 7.25. The ACSF (bath application) contained D-APV (50 µM), an NMDA receptor antagonist + DNQX (10 µM), AMPA/Kainate receptor antagonist. Data were analyzed off-line with Clampfit software of pClamp version 10 (Axon Instruments). For statistical evaluation, paired, unpaired Student’s *t*-test, or ANOVA, were employed by using Origin v8.5 (Microcal Software, Northampton, MA, USA). Results were presented as means ± SEM. For assessment of spontaneous and miniature postsynaptic events, 2–3 min of recordings following drug application were sampled at 5 kHz in neurons voltage clamped at −70 mV, and analyzed for frequencies and amplitudes using Mini Analysis software (Synaptosoft, Leonia, NJ, USA). Synaptic events were detected with an adjustable threshold, often set at 10–20 pA and maintained at a constant level in a given neuron. 

### 4.2. In-Vivo Behavioral Experiments

#### 4.2.1. Animals 

BTBR T + Itpr3tf/J (BTBR) mice (aged 11–13 weeks, weighing 30–35 g) were procured from Jackson Laboratory (Boulevard, Bethesda, MD, USA), and were bred in the local central animal facility of the College of Medicine and Health Sciences, United Arab Emirates University. The inbred C57BL/6J (C57) mice (aged 11–13 weeks, weighing 20–25 g) were also obtained from the animal facility at the College of Medicine and Health Sciences, United Arab Emirates University. The animals were kept in a separate air-conditioned room with controlled temperature and humidity (24 ± 2 °C and 55% ± 15%, respectively), 12 h light/dark cycle, and ad libitum to food and water. All the experiments were performed between 9.00 a.m. and 3.00 p.m. 

#### 4.2.2. Drugs

The test compound CUR (25, 50, and 100 mg/kg, i.p.), the reference acetylcholinesterase drug donepezil (DOZ, 1 mg/kg, i.p.) hydrochloride, and the CNS-penetrant methyllycaconitine (MLA, 1 µg/kg, i.p.) were purchased from Sigma-Aldrich (St. Louis, MO, USA). The assay kits for superoxide dismutase (SOD) and catalase (CAT) were purchased from Cayman Chemical (Ann Arbor, MI, USA). 

#### 4.2.3. Animal Groups and Drug Treatments 

All the mice were acclimatized for one week before the start of the experiment, and subsequently randomly divided into eight groups of seven mice each. Total duration of the experiment was 21 days, the daily intraperitoneal (i.p.) chronic treatment started one week before the behavioral experiments and continued until the sacrifice. The doses as well as saline were injected i.p. 30–45 min before commencement of the behavioral experiments each day. The compounds were dissolved in physiological saline before administration and the volume was normalized according to body weight (10 mL/kg). Group 1, C57 mice injected with saline served as control. Group 2, BTBR mice treated with saline served as autistic control. Groups 3–5, BTBR mice received i.p. injections of different doses of CUR (25, 50, and 100 mg/kg), respectively. Group 6, BTBR mice were injected with reference drug DOZ (1 mg/kg, i.p.), Group 7, BTBR mice received co-injection of CUR (50 mg, i.p.) and MLA (1 µg/kg) for abrogation studies, and Group 8, BTBR mice received i.p. injections of MLA (1 µg/kg). Doses of CUR, DOZ, and MLA were selected according to previous experimental protocols C57 mice as well as BTBR mice [10,38,48,53,54,55,56,57,58,59,60,61,62,63,64,65,66,67,68,69]. In these studies, a dose range of 15–125 mg/kg was used to assess several in vivo effects of CUR in mice. For instance, chronic administration of CUR (15, 30, and 60 mg/kg, p.o.) was found to significantly attenuate vincristine-induced neuropathy in mice which may be due to its multiple actions including antinociceptive, calcium inhibitory, and antioxidant effect [64]. 

Given the poor bioavailability of CUR, a dose range of 25–100 mg/kg CUR was used in the current series of in vivo experiments. In addition, and in numerous previous studies from our group, DOZ (1 mg/kg, i.p.) was proven to show enhancing effects of cognitive functions of BTBR mice [9,10,48,53,55,56,57]. Therefore, a dose of 1 mg/kg of DOZ was selected in the current in vivo behavioral assessments. 

Following behavioral tests, i.e., on day 21 of systemic treatment, all animals were sacrificed. The skull was removed carefully, brains were removed, and both hemispheres were separated on ice plate. The hippocampus and cerebellum were isolated from brain and were frozen immediately with liquid nitrogen for further biochemical assays. 

#### 4.2.4. Behavioral Tests

##### Three Chamber Paradigm (TCP) 

TC test was performed according to previously described protocols [48,53,56,57,62,63]. Briefly, the cage consisted of three chambers, wherein the center chamber had two square shaped openings with doors, which provided access to left and right chambers. Two plastic round wired cages were used to separate the stranger mice from experimental mouse. Following habituation in that plastic wire cage for 30 min and 1 day prior to the commencement of experiments, the total duration of one trial was 30 min, consisting of two 5 min sessions and two 10 min sessions. During the first 5 min of habituation session, the test mouse was placed in the center chamber with no access to other two chambers. For the second 5 min session, it was allowed to access all three chambers by opening the doors. Following habituation and before starting the first 10 min of test session, a stranger mouse was placed in a wire cage in one side of the chamber (same gender and age as the test mouse but with no previous contact) referred to as the novel mouse (NM), meanwhile in the opposite chamber an empty wire cage was placed, representing the novel object (NO). The position of the stranger mice was altered regularly to avoid side preferences. In this session, the test mouse was allowed to access all three chambers for 10 min. This was followed by the second 10 min test session immediately by placing a second stranger mouse in the empty wire cage. In this context, the first stranger mouse was referred to as the familiar mice (FM) and the second stranger mouse was referred to as the novel mouse (NM). A similar pattern of time duration was counted as in first 10 min test session. The entire experiment was recorded and the duration for each session was calculated accordingly, using EthoVision^®^ Software (Noldus, Netherlands). To allow the direct comparison of social behavior of the treated groups, the sociability index (SI) and social novelty preference index (SNI) were evaluated. The SI was calculated as [Time exploring NM—Time exploring NO]/[Time exploring NM + Time exploring NO]; while SNI was calculated as [Time exploring NM—Time exploring FM]/[Time exploring NM + Time exploring FM]. The chambers were thoroughly cleaned with a tissue dampened with 70% (volume/volume; vol/vol) alcohol to remove the odor after each mouse completed sociability and social novelty tests. 

##### Open Field Test 

To analyze the effect of ST-713 on locomotion and anxiety behaviors in animals, an open field test (OFT) test was carried out. In addition to locomotor activity, the OFT is usually used to measure anxiety-like behaviors in experimental rodents [53,57,63]. The test provides a unique opportunity to systematically assess novel environment exploration, general locomotor activity, and provide an initial screen for anxiety-related behavior in experimental rodents. The OFT box consisted of a square box (45 × 45 × 30 cm). A 23 cm × 23 cm area in the center region was defined as central arena, the remaining was defined as periphery area. The mice with higher anxiety degree prefer to stay closer to the walls of the box and spend less time in the center. Whereas increased time spent in the central area indicates low anxiety level and high exploratory behaviors [53,57,63]. The first 5 min of the experiment was considered as habituation, followed by a 10 min test session. The total distance moved in the whole arena, time spent in the center and in the periphery were recorded for 10 min using a charge-coupled device camera-assisted motion tracking apparatus and software (EthoVision 3.1, Noldus Information Technology, the Netherlands) (Table 2). After each mouse has completed the test, the OF chamber was cleaned thoroughly with 70% (volume/volume; vol/vol) alcohol. 

#### 4.2.5. Biochemical Assessments

##### Brain Tissue Collection 

The hippocampus was homogenized in a KCl buffer (Tris-HCl, 10 mM NaCl, 140 mM KCl, 300 mM EDTA, and 0.5% of 1 mM Triton-X-100) at pH 8.0 supplemented with protease and phosphatase inhibitor. The homogenate was centrifuged at 10,000 rcf for 30 min at 4 °C. The supernatant was used according to previously described experimental protocols from our laboratories for the estimation of oxidative stress markers, namely catalase (CAT) and superoxide dismutase (SOD) [48,49,53,56]. 

##### Assessment of Antioxidant Enzymes Activity 

The activities of endogenous antioxidant enzymes superoxide dismutase (SOD) and catalase (CAT) were measured using commercially available assay kits purchased from Cayman Chemicals Co., Ann Arbor, MI, USA (catalog number: 706,002 and 707,002). The SOD activity was measured by incubating the test samples or standards (10 μL) in a 96- well plate. Thereafter, xanthine oxidase (20 μL) was mixed to each well in order to initiate the reaction, after that the plate was lightly mixed by shaking, and the plate covered and incubated at room temperature for 30 min. Absorbance was noted at 450 nm by a microplate reader. The activity of CAT was measured in a 96-well microplate by adding standards or test samples (20 μL) to the assay buffer (100 μL) and methanol (30 μL). To start the reaction, hydrogen peroxide solution (20 μL) was added and samples were incubated at room temperature for 20 min. To stop the reaction, potassium hydroxide (30 μL) was added to each well and further catalase purpald (30 μL) and catalase potassium periodate (10 μL) were added to the plate and kept on a shaker for mixing for 5 min at room temperature. The absorbance was recorded at 540 nm with a VersaMax™, tunable microplate reader (Molecular Devices, San Jose, CA, USA). The activities of SOD and CAT were represented as U/mL and nmol/min/mL, respectively [49,50,65,66,67,68,69,70]. 

### 4.3. Statistics

For behavioral studies and biochemical assessments, data were expressed as means ± SEM. The data were analyzed for normality by assessing the sample distribution or skewness (−1.8 to +1.8 considered normally distributed). After the results had passed the tests for normality, the effects of CUR were analyzed using two-way analysis of variance (ANOVA) with dose of drugs and animals as the between-subjects factor, and post hoc comparisons were performed with Tukey’s test in case of a significant main effect. For statistical comparisons, the software package SPSS 25.0 (IBM Middle East, Dubai, United Arab Emirates) was used. The *p* values less than 0.05 were considered statistically significant.

## 5. Conclusions

Taken together, the observed results indicate that CUR potentiates α7-nAChRs in native central nervous system neurons, mitigates disturbed oxidative stress levels and alleviate ASD-like features in BTBR mice used as an idiopathic rodent model of ASD. The results of above-mentioned in vitro, in vivo, and related clinical studies strongly suggest that deficits in the α7-nAChR are closely linked to the development of ASD, and stimulation of α7-nAChRs has potential beneficial effects in the future pharmacological management of ASD. Considering the significant variable impeding the therapeutic applicability of CUR, e.g., problems associated with its relatively low solubility, poor oral bioavailability, and chemical instability at neutral and slightly alkaline mediums [34], further assessments of the therapeutic potential role of CUR still must be certified for use in humans, and additional research is also required to help elucidate optimal therapeutic dosage, treatment duration, and the efficacy of CUR in neuropsychiatric disorders, e.g., ASD.

## Figures and Tables

**Table 1:** Effects of curcumin and donepezil systemic pretreatment on anxiety levels and locomotor activity in BTBR mice.

Treatment Group	Time Spent in Center (s)	Time Spent in Periphery (s)	Total Distance Travelled (cm)
SAL (C57)	30.64 ± 3.72	569.36 ± 5.31	2522 ± 187.75
SAL (BTBR)	62.75 ± 4.18 **	537.25 ± 6.19 **	4166 ± 168.14 **
CUR (25 mg/kg)/BTBR	59.04 ± 4.54	540.96 ± 10.85	3897 ± 128.76
CUR (50 mg/kg)/BTBR	59.91 ± 5.57	536.44 ± 12.82	3936 ± 171.15
CUR (100 mg/kg)/BTBR	58.07 ± 5.79	540.87 ± 9.27	3787 ± 330.29
DOZ (1 mg/kg)/BTBR	43.64 ± 3.37 ^#^	556.36 ± 6.95	4066 ± 183.01
CUR(50 mg/kg) + MLA (1µg/kg)/BTBR	57.61 ± 4.97	542.39 ± 7.94	3905 ± 152.65
MLA (1µg/kg)/BTBR	59.00 ± 4.65	541.00 ± 8.19	4149 ± 147.64

BTBR mice demonstrated elevated impulsive attitude and deficits in cognition as well as locomotor activity behaviors that were significantly increased compared to C57 mice. CUR (25, 50, or 100 mg/kg, i.p.) or DOZ (1 mg/kg, i.p.) were administered chronically for 21 days. CUR (25, 50, and 100 mg/kg, i.p.) failed to significantly attenuate the increased time spent in the central arena. However, DOZ (1 mg/kg, i.p.) significantly attenuated the increased time spent in the central arena. Also, CUR (25, 50, or 100 mg/kg, i.p.) or DOZ (1 mg/kg, i.p.) failed to alter the increased total distance travelled as well as the time spent in the periphery in BTBR mice in the OF test. Data are expressed as the means ± SEM (*n* = 7). ** *p* < 0.001 vs. C57 mice. ^#^ *p* < 0.05 vs. saline-treated BTBR mice. The effects of CUR on locomotor activity as well as anxiety-like behaviors were analyzed using two-way analysis of variance (ANOVA) with dose of drugs and animals (either BTBR or C57 mice) as the between-subjects factor, and post hoc comparisons were performed with Tukey’s test in case of a significant effect.

**Table 2:** Curcumin and donepezil mitigated levels of oxidative stress markers in the cerebellum and the hippocampus of treated BTBR mice.

		BTBR						
			CUR					
	C57 (SAL)	(SAL)	25 mg/kg	50 mg/kg	100 mg/kg	50 mg/kg + MLA (1 µg/kg)	MLA (1 µg/kg)	DOZ (1 mg/kg)
**SOD**								
*Cerebellum*	50.95 ± 1.89	36.42 ± 2.19 ***	38.24 ± 2.05	36.16 ± 1.73	39.41 ± 3.04	36.91 ± 1.38	37.67 ± 1.26	36.52 ± 1.73
*Hippo-campus*	49.47 ± 1.52	37.70 ± 1.61 ***	39.55 ± 1.67	49.36 ± 0.5 ^##^	50.37 ± 1.55 ^##^	48.61 ± 3.24	38.63 ± 1.45	36.95 ± 2.67
**CAT**								
*Cerebellum*	159.43 ± 6.92	127.95 ± 3.37 *	128.30 ± 4.07	151.11 ± 5.47 ^#^	149.82 ± 4.72 ^#^	148.07 ± 5.10 ^#^	127.95 ± 3.37	133.95 ± 5.21
*Hippo-campus*	26.53 ± 4.90	106.58 ± 3.01 *	104.13 ± 7.54	105.50 ± 3.77	108.11 ± 5.61	104.08 ± 3.97	103.38 ± 6.23	105.58 ± 3.12

Superoxide dismutase (SOD) and catalase (CAT) were assessed in two different brain parts of treated BTBR mice, namely the cerebellum and the hippocampus. BTBR mice showed a significant decrease in SOD and CAT compared to C57 mice. Effects of systemic administration of CUR (25, 50, and 100 mg/kg, i.p.) was assessed in C57 and BTBR mice. CUR (50 and 100 mg/kg, i.p.) significantly increased the reduced levels of SOD in the hippocampus, but failed to increase the decreased levels of SOD in the cerebellum of treated BTBR mice. CUR (50 and 100 mg/kg, i.p.) significantly increased the reduced levels of CAT in the cerebellum, but failed to increase the decreased levels of CAT in the hippocampus. *** *p* < 0.001 vs. C57 mice. ^#^ *p* < 0.05 vs. saline-treated BTBR mice. ^##^ *p* < 0.05 vs. saline-treated BTBR mice. The effects of CUR were analyzed using two-way analysis of variance (ANOVA) with dose of drugs and animals (either BTBR or C57 mice) as the between-subjects factor, and post hoc comparisons were performed with Tukey’s test in case of a significant effect.

## Supplementary Materials

The following are available online at https://www.mdpi.com/article/10.3390/ijms22147251/s1.

## Abbreviations

Autistic spectrum disorder, ASD; Nicotinic acetylcholine receptors, nAChRs; Attention-deficit hyperactivity disorder, ADHD; Black and Tan BRachyury, BTBR; Superoxide dismutase, SOD; Catalase, CAT; Acetylcholinesterase inhibitor, AChEI; Donepezil, DOZ; Curcumin, CUR; Methyllycaconitine, MLA; Artificial cerebral spinal fluid, ACSF; D-(-)-2-Amino-5-phosphonopentanoic acid, D-APV; 6,7-Dinitroquinoxaline-2,3-dione, DNQX; MB, TCP, sociability index, SI; social novelty preference index, SNI; Novel object, NO; Novel mouse, NM; Familiar mouse, FM; Three chamber Paradigm, TCP; Open Field Test, OFT.
