# Supplementary material for: Curcumin Potentiates α7 Nicotinic Acetylcholine Receptors and Alleviates Autistic-Like Social Deficits and Brain Oxidative Stress Status in Mice
Source: Int J Mol Sci. 2021 Jul 6;22(14):7251. doi: 10.3390/ijms22147251 (PMC8303708; doi:10.3390/ijms22147251)
Supplement: Supplementary file 1 [file ijms-22-07251-s001.zip › ijms-1265426-SI.pdf]

Supplementary Materials:

The following are available online at www.mdpi.com/xxx/s1.

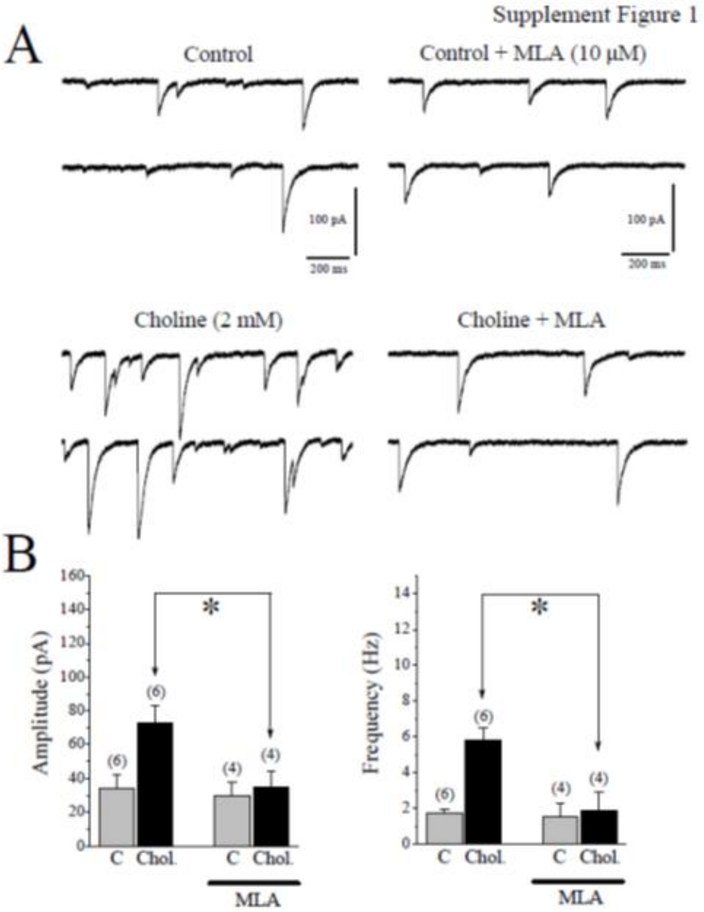

Figure S1. The effect of methyllycaconitine on choline-induced enhancement of GABAA receptor-mediated spontaneous synaptic events in CA1 pyramidal neurons. Whole-cell recordings were performed using CsCl-based electrode solution at a holding potential of −70 mV. (A) On the left, application of choline (2 mM) for 30 sec increased the amplitudes and frequencies of spontaneous inhibitory postsynaptic currents (sIPSCs; $n = 6$). On the right, in another cell, choline-induced enhancements of sIPSCs were reduced significantly after co-application of choline and methyllycaconitine (10 μM). (B) Summary of the effects of methyllycaconitine (10 μM) on choline-induced responses. The averaged amplitudes (on the left) and the frequencies (on the right) of sIPSCs were presented before (control C, gray bars) and after (black bars) choline (Chol. 2 mM) application. For comparison, the effect of choline on the GABAA receptor-mediated sIPSCs is shown in the absence (same set of neurons used in left panel of the Figure 2) and the presence of methyllycaconitine. Bars represent the means ± S.E. of four to six experiments (* $p < 0.05$ vs. control; ANOVA). C, control; Chol., choline; and MLA, methyllycaconitine.
